# Disseminated intravascular coagulation as a complication of bursitis: angiogenesis and repetitive bleeding as potential factors for disseminated intravascular coagulation: a case report

**DOI:** 10.1186/s13256-021-02773-x

**Published:** 2021-04-10

**Authors:** Taisuke Hamada, Tasuku Nishihara, Yosuke Mizuno, Teruki Kidani, Hiroki Tokiwa, Naoki Abe, Keisuke Sekiya, Sakiko Kitamura, Yasushi Takasaki, Hiromasa Miura, Toshihiro Yorozuya

**Affiliations:** 1 Department of Anesthesia and Perioperative Medicine, Ehime University Graduate School of Medicine, Toon, Ehime 791-0295 Japan; 2 Division of Diagnostic Pathology, Ehime University Hospital, Toon, Ehime Japan; 3 Department of Orthopaedic Surgery, Ehime University Graduate School of Medicine, Toon, Ehime Japan

**Keywords:** Disseminated intravascular coagulation, DIC, Fibrinolytic phenotype, Bursitis, Chronic inflammation, Angiogenesis, Intratumoral bleeding

## Abstract

**Background:**

Malignant tumors, such as acute leukemia and solid cancers, frequently cause disseminated intravascular coagulation. However, cases of disseminated intravascular coagulation as a complication of bursitis were not reported previously.

**Case presentation:**

A 72-year-old Japanese woman was scheduled to undergo resection of a rapidly growing subcutaneous tumor-like lesion on her left back. Preoperative blood tests suggested disseminated intravascular coagulation. The resected lesion was cystic tumor containing a hematoma. After the operation, the patient completely recovered from disseminated intravascular coagulation, indicating that disseminated intravascular coagulation in this case was caused by the tumor. Pathological examination of the resected tumor revealed considerable fibrin deposition and angiogenesis on the cyst wall, which was presumably a response to inflammation and indicated presence of repetitive intratumoral bleeding, subsequently leading to a diagnosis of chronic hemorrhagic bursitis.

**Conclusions:**

Clinicians should note that, despite being benign, soft-tissue tumors accompanied by inflammation with angiogenesis and repetitive intratumoral bleeding can cause disseminated intravascular coagulation, albeit rarely.

**Supplementary Information:**

The online version contains supplementary material available at https://doi.org/10.1186/s13256-021-02773-x.

## Background

Disseminated intravascular coagulation (DIC) is a pathological condition wherein the coagulation system is activated systemically and persistently in the presence of underlying diseases, such as bacterial infection, leukemia, and malignant neoplasms. Intravascular clots formed throughout the body occlude small blood vessels, which cause multiple organ failure that may be critical in some cases [1–3]. Although each specialized doctor encounters different underlying diseases, for example, sepsis by bacterial infection for anesthesiologists and intensivists, trauma for emergency physicians, malignant tumors for oncologists, and leukemia for hematologists, DIC itself occurs frequently in any field. However, DIC cases as a complication of benign soft-tissue tumors have been rare, with no reports regarding DIC as a complication of bursitis. Accordingly, we describe a case involving DIC with a fibrinolytic phenotype as a complication of bursitis.

## Case presentation

**Fig. 1:**
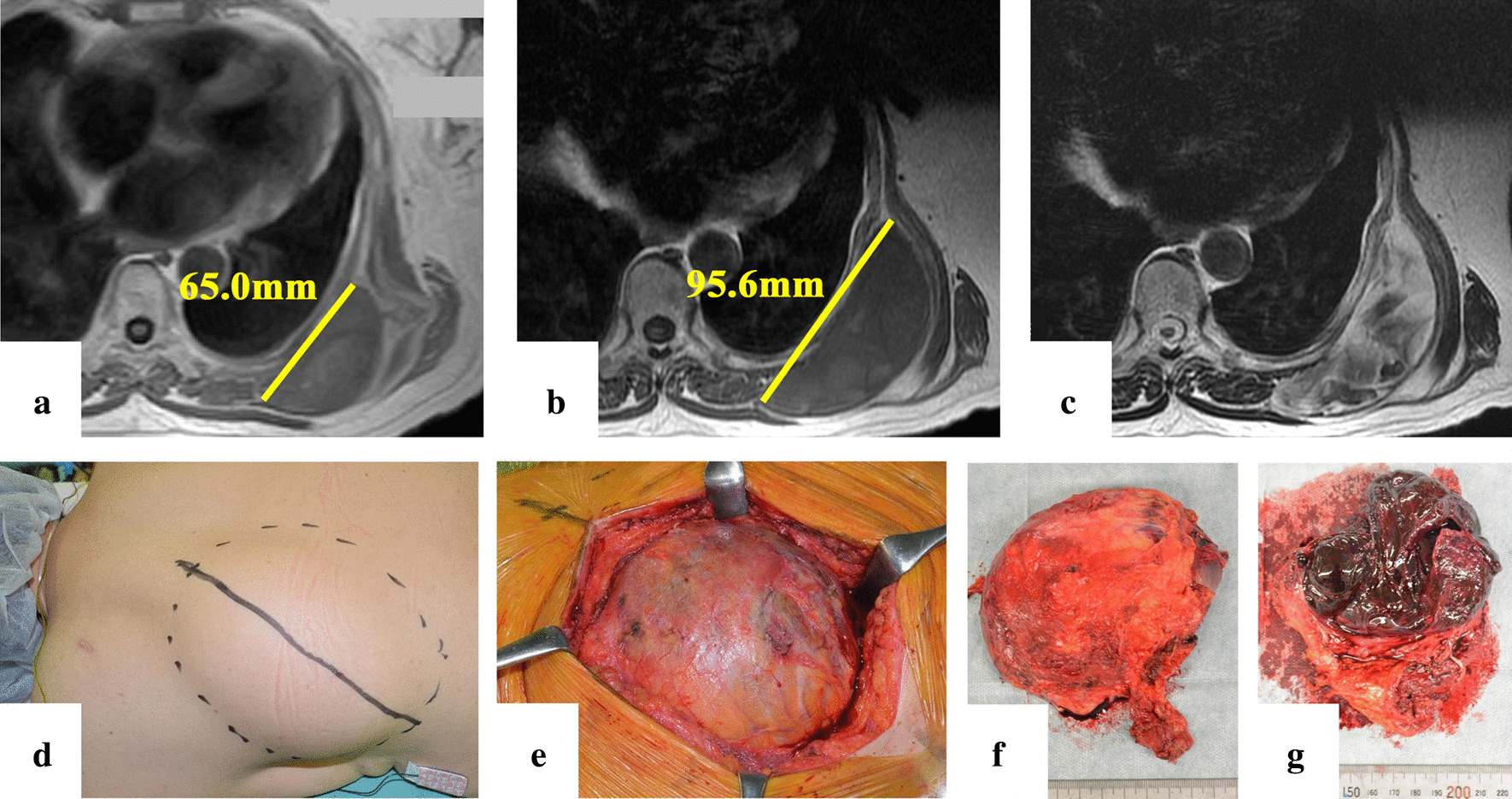
**a** T1-weighted magnetic resonance imaging (MRI) image 2 months before the surgery. The tumor size was 65 mm. **b** T1-weighted MRI image 2 days before the surgery. The inside of the tumor exhibited low-signal intensity, while an increase in size was observed (95.6 mm) compared with that 2 months before. **c** T2-weighted image of the same layer shown in **b**. The inside of the tumor exhibited heterogeneous high-signal intensity. **d** Protuberant tumor on the patient’s back. **e** and **f** Resected tumor and a membrane with a smooth surface covering the tumor. **g** Section of the resected tumor. The tumor was filled with blood clots

A 72-year-old Japanese woman perceived protrusion on her back. She had a history of appendectomy, diabetes, cholecystectomy, hypertension, and transverse colectomy. At approximately 40 days before the surgery, a subcutaneous tumor-like lesion 65.0 mm in diameter in the patient’s left back was identified through magnetic resonance imaging (MRI; Fig. 1a). The tumor-like lesion was rapidly growing, and she was scheduled to undergo elective resection.

She was admitted to our institution 2 days before the surgery (postoperative day minus 2 [POD-2]). Body temperature was 36.7 °C. Any symptoms indicating infection were not observed. MRI revealed an increase in size to 95.6 mm in diameter (Fig. 1b). Hemorrhagic bursitis, which is one of the differential diagnoses, was suspected based on the following MRI findings: low-signal intensity and partly high-signal intensity on T1-weighted images (Fig. 1b) and heterogeneous high-signal intensity on T2-weighted images (Fig. 1c) inside the majority of the tumor consistent with intratumoral bleeding. We consulted a hematologist because preoperative blood examination revealed a decrease in platelet count and fibrinogen level and an increase in D-dimer, fibrin/fibrinogen degradation product (FDP), indicating DIC. DIC with a fibrinolytic phenotype was diagnosed by abnormal results of additional blood examination: increase in thrombin–antithrombin complex, and plasmin-α_2_-plasmin inhibitor complex (PIC) (Additional file 1: Table S1). Any other underlying disease that could have caused DIC, except for the tumor, was not identified. Antifibrinolytic therapy with tranexamic acid (TA) was subsequently started after consulting a hematologist, given that DIC, especially the fibrinolytic phenotype, causes excessive bleeding and hemostatic difficulty during surgery. Accordingly, 2 g/day of TA was administered perioperatively [4]. Moreover, 3 g of concentrated fibrinogen product, 10 units of platelet concentrates, and 4 units of packed red blood cells were transfused before and during the surgery.

After general anesthesia was induced, the patient was placed in the prone position. The skin over the lesion area had been raised by the tumor (Fig. 1d). The tumor adhered to the thoracic wall and surrounding tissues and was partly rich in vasculature. A smooth membrane covered the tumor (Fig. 1e and f), while the inside was filled with hematoma (Fig. 1g). The condition of the fibrinolytic system was analyzed using thromboelastometry (ROTEM Delta) during the surgery. A coagulation test was also performed to maintain a fibrinogen level of > 150 mg/dl. Thromboelastometry results during the surgery revealed an improvement in enhanced fibrinolysis resulting from continuous TA administration and fibrinogen transfusion. The surgery, which lasted for 53 minutes, was completed without critical bleeding or complications, during which the amount of bleeding was 370 ml. After the procedure, the patient was transferred to the surgical ward until she was discharged.

**Fig. 2:**
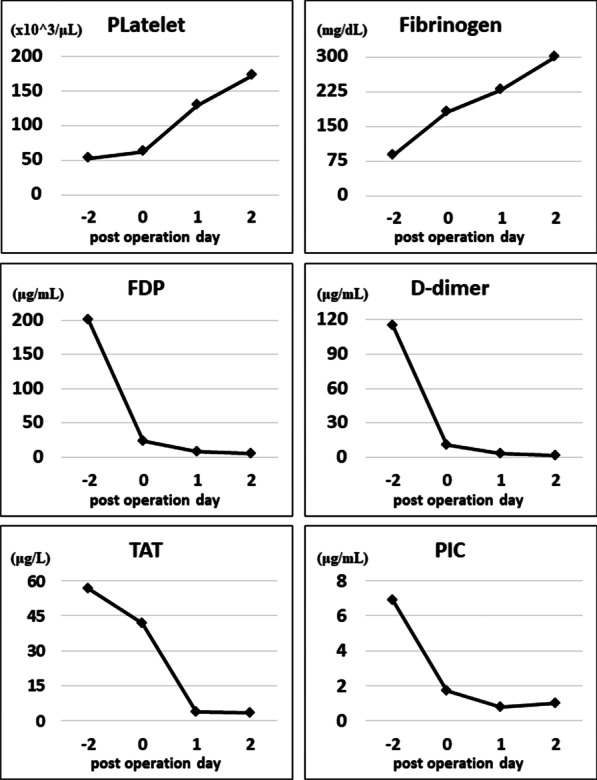
Factors related to disseminated intravascular coagulation are shown in the graph. Disseminated intravascular coagulation was ameliorated after the operation

Blood examination was performed to evaluate the status of DIC at POD 0 (after the operation), 1, and 2 (Fig. 2 and Additional file 1). FDP, D-dimer, and PIC levels had decreased after antifibrinolytic therapy with TA. After the operation, the patient completely recovered from DIC, indicating that DIC in this case was caused by the tumor.

**Fig. 3:**
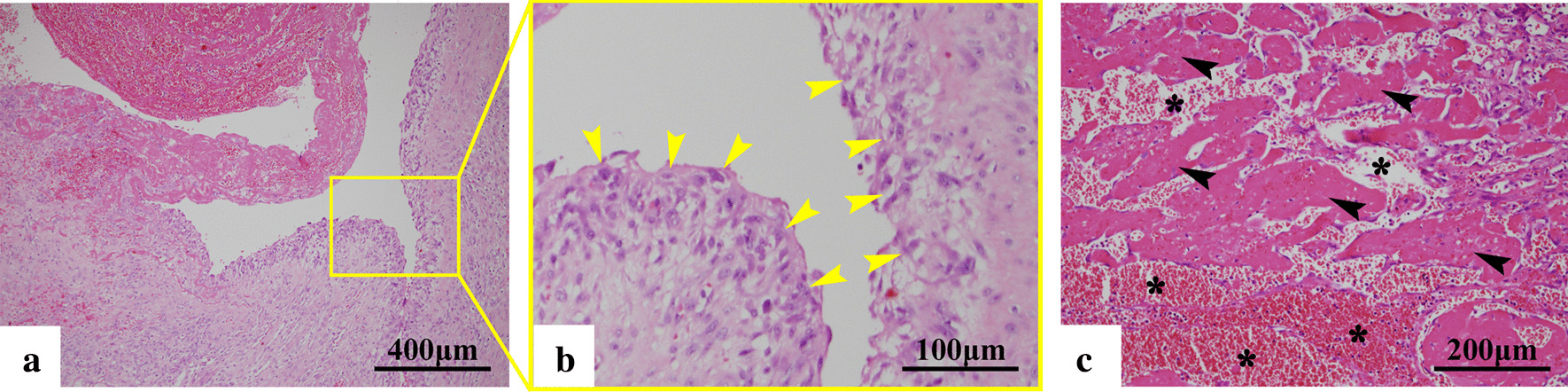
Pathological images of the resected tumor. **a** The cyst wall under low magnification. **b** Cell aggregates in the cyst wall comprising synovial cells (arrowhead). **c** A portion of the cyst wall under high magnification. Extensive fibrin deposition (asterisks) and neovascularization (arrowheads) were observed

The pathological reports on POD 10 showed that the tumor was a cyst containing a hematoma (Fig. 3a) and that synovial cells comprised the cyst wall (Fig. 3b). The cyst wall was abundant in fibrin deposition (Fig. 3c, asterisk) and exhibited neovascularization (Fig. 3c, arrowhead), which was suspected as bleeding and reactive angiogenesis to the inflammation. Accordingly, a pathological diagnosis of chronic hemorrhagic bursitis was established.

## Discussion

A synovial bursa, which is a flat latent cavity containing synovial fluid, acts as a cushion during muscle, tendon, and bone movement. In general, therapies for bursitis include rest, nonsteroidal antiinflammatory agents, and/or local injection of steroids. However, surgical resection could be considered in some cases, for example, compression of nearby organs and nerves. Although the cause for the current case remained unknown, intratumoral bleeding had already been observed during her first hospital visit, which caused the increase in tumor size, and elective operation was planned. DIC encourages further intratumoral bleeding, which also accelerates the development of DIC, thereby creating a negative cycle. DIC is occasionally life threatening, and therapies for such include elimination of the underlying disease. The present case exhibited indications for surgery also upon the presence of DIC.

Based on the blood examinations, a diagnosis of DIC with a fibrinolytic phenotype was established in the present case. Based on the guidelines of some committees [5, 6], we suspected DIC from the decreased fibrinogen and platelet count. In addition to these routine preoperative blood examinations, D-dimer and FDP tests that orthopedic surgeons order routinely to check for deep vein thrombosis were useful for the diagnosis of DIC in this case.

The activation of the blood coagulation pathways is triggered by tissue factors from monocytes/macrophages stimulated by endotoxins in the case of bacterial infection or from tumor cells in the case of malignant tumors, such as acute leukemia and solid cancers [1, 3, 7]. Such pathophysiologies are representative of DIC. Aside from the aforementioned cases, DIC as a complication of hemangioma also exists as represented by Kasabach–Merritt syndrome (KMS). The generally presumed pathophysiology of KMS is that platelets become trapped by an abnormally proliferating endothelium in the hemangioma [8, 9], leading to platelet activation with secondary consumption of clotting factors [10].

The pathophysiology of the present case would differ from those of above but may be more simple. First, the possibility that this case was a hemangioma was excluded, and a diagnosis of hemorrhagic bursitis was established based on the following reasons: (1) a lobular structure, which is usually observed in cases of hemangioma, had not been found in the area of vascular proliferation on histopathological examination, (2) the genesis of hemangioma from the synovial bursa had not been reported, and (3) this diagnosis was consistent with that by MRI. According to the results of the histopathological examination, a substantial amount of hematoma and extensive neovascularization with fibrin deposition had been observed inside the tumor and on the cyst wall, respectively. It was presumed that vascular proliferation was reactive angiogenesis to inflammation. These findings indicate that hemorrhage and inflammation had been present and that platelets and coagulating factors were consumed inside the tumor, resulting in DIC. Nevertheless, it should be notable that the bleeding area was just inside the lesion and was 10 cm in diameter.

Angiogenesis, a normal physiologic reaction of a living body, occurs in an inflamed site. However, such neovascularization is sometimes vulnerable. In such cases, it may bleed easily in the synovial bursa, considering that it moves and is scraped for cushioning. In cases wherein bleeding inside the solid tumor is observed, compression from the surrounding tissues may provide astriction. However, when the bleeding unfortunately occurs inside the synovial bursa, that is, inside the cavity as in the present case, astriction may not work. The present case indicated that chronic inflammation accompanied by angiogenesis and repetitive bleeding could perhaps be a potential factor for DIC despite being a local lesion. In such cases, surgical resection should be selected for the prevention or treatment of DIC.

## Conclusion

This is the first report to describe the case of DIC as a complication of chronic hemorrhagic bursitis. The tumor-like lesion was a cyst containing a hematoma, while extensive angiogenesis and fibrin deposition were observed on the cyst wall. Despite being benign, the lesion accompanied by inflammation with angiogenesis and repetitive intratumoral bleeding can cause DIC.

## Supplementary Information

**Additional file 1: Table S1.** Blood examination results. Blood examination at postoperation day (POD)-2 indicated DIC with a fibrinolytic phenotype, which ameliorated after the operation

## Data Availability

All data generated or analyzed during this study are included in this published article and its supplementary information files.

## Abbreviations

DIC: Disseminated intravascular coagulation
MRI: Magnetic resonance imaging
POD: Post operation day
T1WI: T1-weighted images
T2WI: T2-weighted images
FDP: Fibrin/fibrinogen degradation product
TAT: Thrombin–antithrombin complex
PIC: Plasmin-α_2_ plasmin inhibitor complex
TA: Tranexamic acid
NSAIDs: Nonsteroidal antiinflammatory agents
TF: Tissue factors
KMS: Kasabach–Merritt syndrome
